# Spatial-Temporal Evolution Analysis of Carbon Emissions Embodied in Inter-Provincial Trade in China

**DOI:** 10.3390/ijerph19116794

**Published:** 2022-06-01

**Authors:** Tianrui Wang, Yu Chen, Leya Zeng

**Affiliations:** 1 School of Teacher Education, Nanjing Normal University, Nanjing 210023, China; 2 School of Geography, Nanjing Normal University, Nanjing 210023, China; chenyu@lreis.ac.cn; 3 School of Information and Navigation, Air Force Engineering University, Xi’an 710077, China; zengleya@163.com

**Keywords:** embodied carbon emissions, inter-provincial trade, multi-regional input–output table, network analysis, spatial-temporal evolution analysis

## Abstract

Under the support of Multi-Regional Input–Output (MRIO) analysis, this study constructs the Embodied Carbon Emission Transfer Network (ECETN) using the input–output tables of 42 sectors in 31 provinces of China in 2012, 2015, and 2017 and applies a series of complex network measurement indicators and analysis methods to describe its evolution features. The results show that the embodied carbon emission transfers between provinces generally narrow over time. With its high clustering coefficient and short average path length, ECETN has small-world characteristics and behaves sensitively, and changes in individual provinces can quickly spread and affect the entire system. In addition, the clustering effect and the spatial spillover structural properties of ECETN are explored based on the block model analysis. Finally, Quadratic Assignment Procedure (QAP) is used to analyze and quantify the contribution of provincial structural roles to ECETN, and it is found that spatial adjacency and differences in strength-in, strength-out, and betweenness centrality have significant positive effects, while differences in eigenvector centrality, clustering coefficient have significant negative effects. The restructuring of domestic trade can help achieve national emission reduction. These findings can provide more insights for the government to formulate future development directions and policies to reduce emissions further.

## 1. Introduction

Climate change has become an issue of concern around the world. The Sixth Assessment Report of the Intergovernmental Panel on Climate Change (IPCC) shows that the global climate change problem has already become quite serious [1]. The report clarifies that achieving the goal of limiting global temperature rise to 1.5 °C is still possible, but immediate action needs to be taken [2]. CO_2_, the most significant greenhouse gas, also bears the brunt of widespread attention.

China is an emerging economy and needs to maintain rapid economic development to eliminate poverty and achieve prosperity. China’s energy mix is dominated by fossil fuels, which is difficult to change in the short term, making it particularly challenging to reduce carbon emissions [3,4]. However, China has long attached great importance to the issue of climate change and has made the active response to climate change a significant strategy for national economic and social development [5]. After years of unremitting pursuit and efforts, China has achieved remarkable results in energy conservation and emission reduction, submitted the “Enhanced Actions on Climate Change: China’s Intended Nationally Determined Contributions” to the United Nations in 2015 [5], and pledged to the world at the 2020 United Nations General Assembly to strive to peak CO_2_ emissions by 2030 and work towards achieving carbon neutrality by 2060.

The international community has reached a certain degree of consensus on CO_2_ emission reduction [6]. However, there are still large differences among countries on the division of responsibility for carbon emissions, especially on the attribution of embodied carbon emissions in trade [7,8], and scholars try to assign responsibility for embodied CO_2_ emissions in international trade. Many studies attempt to quantify the CO_2_ emissions embodied in trade, and from these studies, it is found that the percentage of total embodied CO_2_ emissions increases over time [9]. In 2020, China proposed to establish a “dual circulation” development pattern in which domestic economic cycle plays a leading role while international economic cycle remains its extension and supplement. This accelerates the fragmentation of the supply chain, making the spatial separation of production and consumption widespread [6,10]. The level of embodied carbon emission transfers generated by inter-provincial trade in China has exceeded that of international trade [11]. Therefore, the carbon emission transfers caused by inter-provincial trade is also critical in formulating emission reduction policies.

The concept of embodied carbon can be traced back to the working group conference on energy analysis of the International Federation of Institutes for Advanced Studies (IFIAS) in 1974. The conference first introduced the concept of embodied energy to measure the total amount of energy consumed directly and indirectly in producing a product or service, which was later extended to the study of carbon emissions, resulting in the term embodied carbon [12,13]. With the development of economic and trade globalization, the environmental problems caused by trade have become increasingly prominent, and the research on carbon embodied in trade has gradually become a hot topic. For example, Yang et al. (2022) constructed an embodied carbon calculation model in China’s export trade and found that the embodied carbon mainly came from the secondary industry, accounting for more than 90% of the total embodied carbon, while the proportion of embodied carbon in the primary and tertiary industries was relatively low [14]. Li et al. (2021) evaluated the total embodied carbon emissions in China’s exports and 14 sectors from 1992 to 2020 [15]. Based on cross-country panel data, Qayyum et al. (2021) investigate the relationship between economic complexity and embodied carbon emissions [16]. Huo et al. (2021) identified the drivers of emission changes at global and regional scales by quantifying the evolution of carbon emissions in services trade from 2010 to 2018 [17].

The input–output model, founded by Leontief in 1936 [18]. In the 1970s, Leontief pioneered the application of this method to research in environmental protection and obtained the Environmentally Extended Input–Output Analysis (EEIOA) by incorporating environmental data into the economic input–output table [19,20,21]. In the past few years, with the increasingly severe problem of climate change, the Multi-Regional Input–Output (MRIO) model based on EEIOA is widely used in exploring the relationship between human economic activities and climate change [22]. Several scholars used the model to study the embodied carbon emissions in trade in a particular country or major world economy. Li et al. (2020) confirmed the nonlinear relationship between trade volumes and carbon emissions between developed and emerging economies using the MRIO model [23]. Long et al. (2018) used the MRIO model to empirically analyze and compare the direct and total CO_2_ emission intensities of China and Japan from 2000 to 2014 and bilateral economic activities such as imports, exports, production, and consumption [24]. Based on the MRIO model, Zhang et al. (2021) investigated the global pollution paradise phenomenon in 43 countries and 56 major sectors from 2000 to 2014 [25]. Zhang et al. (2019) analyzed the embodied carbon emissions in trade within Brazil, Russia, India, China, and South Africa (BRICS) and between the BRICS group and other economies [26].

The above studies only consider the embodied emissions in trade from a global and national perspective, ignoring the differences between different regions within a country. Studying the embodied carbon emissions in trade between different regions or provinces is necessary. For example, Zhou et al. (2018) estimated the embodied carbon emissions of various regions in China from 2002 to 2012, studied how they transferred through major regions and key sectors [27]. Yuan et al. (2022) calculated the carbon footprints of nine provinces in the Yellow River Basin of China and estimated the embodied carbon emission transfers between provinces and industrial sectors [28]. Lv et al. (2019) conducted an empirical study on the inter-provincial embodied carbon emission transfers in China and analyzed the spatial correlation and influencing factors [29].

In order to profoundly investigate the relational characteristics within the system, complex network theory and its metrics provide a new approach to analyzing the system and its individuals from a global perspective [30]. In the past few years, with the continuous research of complex network theory, it is widely used in the research of energy [31], economy [32], transportation [33,34,35], and other disciplines, and some scholars also apply it to the study of embodied carbon emission transfers. Based on the input–output tables of China from 2002 to 2015, Wang et al. (2021) constructed six embodied carbon emission flow networks containing 30 sectors and analyzed them by complex network metrics to obtain the overall structural characteristics, key sectors, and critical flow paths [36]. Li et al. (2020) used a series of network tools to describe the evolution characteristics of the global carbon flow network from 1995 to 2011 [6].

This paper combines MRIO and complex network analysis methods to empirically investigate the carbon emission transfers embodied in inter-provincial trade in China. First, using inter-provincial trade input–output tables and provincial CO_2_ emissions data, we account for the embodied carbon emissions of 42 sectors in 31 Chinese provinces in 2012, 2015 and 2017. Second, the Embodied Carbon Emission Transfer Network (ECETN) is constructed by utilizing the carbon emission transfer relationships among provinces. The network topological structure characteristics, province roles, clustering characteristics and spatial spillover structure characteristics are evaluated and analyzed using complex network theory. Third, correlation and regression analyses using the Quadratic Assignment Procedure (QAP) are employed to identify the interlinkage and potential drivers between provincial roles and embodied carbon emission transfers. Finally, we present policy recommendations based on our results for coordinating inter-provincial emission reductions and cutting carbon emissions even more. We explore the complex interaction between economic trade and the environment from a systemic perspective, taking into consideration both the independence of the subjects of each province and their interconnectedness, and we concentrate on both economic activities and their environmental impacts. In addition, by discussing China’s CO_2_ emissions at the provincial level, we can help understand the main contradictions in each province, formulate localized emission reduction policies, and achieve China’s carbon peak target earlier.

In addition to this introduction, the rest of the paper is organized as follows. In Section 2, the embodied carbon emissions in inter-provincial trade are measured based on MRIO analysis and construct the ECETN. In addition, complex network theory, QAP analysis, and the data used are described. Section 3 presents the results of the empirical study. Conclusions as well as policy implications are given in Section 4.

## 2. Research Methods and Data

### 2.1. MRIO Analysis

In this study, the 31 provinces of China are studied, so we use the MRIO model. The basic structure of the Chinese MRIO table used in this study is shown in Table 1. There are *m* provinces in the model, each consisting of *n* intermediate use sectors and *k* final use categories. Where $z_{ij}^{sr}$ denotes the intermediate use capital flow from sector *i* in province *s* to sector *j* in province *r*, and $z_{ij}^{sr}$ is an element of the intermediate use matrix *Z*. $y_{i\mu }^{sr}$ represents the final-use capital flow of sector *i* in province *s* used in category μ of province *r* and is an element of the final use matrix *Y*. $x_{i}^{s}$ represents the total capital output/input of sector *i* in province *s* and is an element of the total output/input matrix *X*. $e_{i}^{s}$ denotes the export capital flow of sector *i* in province *s* and is an element of the export matrix *E*. $I_{i}^{s}$ denotes the import capital flow of sector *i* in province *s* and is an element of the import matrix *I*. $I_{\mu }^{f,s}$ is the capital flow of import for the final use of category μ in province *s*. $v_{i}^{s}$ represents the value of the initial capital invested in sector *i* of province *s* and is an element of the initial input capital matrix *V*. $d_{i}^{s}$ is the direct carbon emissions from sector *i* in province *s* during the production process.

In Table 1, we can get the following equilibrium relations in the direction of rows:

(1) $$\sum_{r}\sum_{j}z_{ij}^{sr}+\sum_{r}\sum_{\mu }y_{i\mu }^{sr}+e_{i}^{s}=x_{i}^{s}$$

In the direction of the columns, we can obtain the following equilibrium relations:

(2) $$\sum_{s}\sum_{i}z_{ij}^{sr}+v_{j}^{r}+I_{j}^{r}=x_{j}^{r}$$

Using matrix operations, we can rewrite Equation (1) as follows:

(3) X=Z+E+Y

By introducing the intermediate input coefficient matrix *A*, we can obtain Z=AX. The basic element $a_{ij}^{sr}=z_{ij}^{sr}/x_{j}^{r}$ of matrix *A* is the intermediate input coefficient of sector *i* in province *s* to sector *j* in province *r*. It represents the capital required to be invested in sector *i* in province *s* for each unit of product produced in sector *j* in province *r*. Based on this, Equation (3) can be rewritten as:

(4) X=AX+E+Y

(5) $$\left(\begin{array}{c} X^{1}\\ X^{2}\\ \vdots \\ X^{m} \end{array}\right)=\left(\begin{array}{cccc} A^{11} & A^{12} & \cdots & A^{1m}\\ A^{21} & A^{22} & \cdots & A^{2m}\\ \vdots & \vdots & \ddots & \vdots \\ A^{m1} & A^{m2} & \cdots & A^{mm} \end{array}\right)\left(\begin{array}{c} X^{1}\\ X^{2}\\ \vdots \\ X^{m} \end{array}\right)+\left(\begin{array}{c} E^{1}\\ E^{2}\\ \vdots \\ E^{m} \end{array}\right)+\left(\begin{array}{c} Y^{1}\\ Y^{2}\\ \vdots \\ Y^{m} \end{array}\right)$$

In MRIO analysis, the import and export of provinces are usually not considered, then Equation (5) can be rewritten as:

(6) $$\left(\begin{array}{c} X^{1}\\ X^{2}\\ \vdots \\ X^{m} \end{array}\right)=\left(\begin{array}{cccc} A^{11} & A^{12} & \cdots & A^{1m}\\ A^{21} & A^{22} & \cdots & A^{2m}\\ \vdots & \vdots & \ddots & \vdots \\ A^{m1} & A^{m2} & \cdots & A^{mm} \end{array}\right)\left(\begin{array}{c} X^{1}\\ X^{2}\\ \vdots \\ X^{m} \end{array}\right)+\left(\begin{array}{c} Y^{11}+\sum_{j\neq 1}Y^{1j}\\ Y^{22}+\sum_{j\neq 2}Y^{2j}\\ \vdots \\ Y^{mm}+\sum_{j\neq m}Y^{mj} \end{array}\right)$$

Further, the output of each province can be written as:

(7) $$X^{s}=A^{s1}X^{1}+A^{s2}X^{2}+\cdots +A^{sm}X^{m}+Y^{ss}+\sum_{j\neq s}Y^{sj}$$

(8) $$X^{s}=A^{ss}X^{s}+Y^{ss}+\sum_{j\neq s}\left(A^{sj}X^{j}+Y^{sj}\right)$$

(9) $$X^{s}={\left(I-A^{ss}\right)}^{-1}\left[Y^{ss}+\sum_{j\neq s}\left(A^{sj}X^{j}+Y^{sj}\right)\right]$$

According to Equation (9), we can get the input–output relationship of each province. The input–output relationship within the province is:

(10) $$R^{ss}={\left(I-A^{ss}\right)}^{-1}\times Y^{ss}$$

The input–output relationship among provinces is:

(11) $$R^{sr}={\left(I-A^{ss}\right)}^{-1}\times \left(A^{sr}X^{r}+Y^{sr}\right)$$

where $R^{ss}$ denotes the matrix of input–output relationships among sectors within province *s*, and $R^{sr}$ denotes the matrix of input–output relationships among sectors from province *s* to province *r*.

### 2.2. Calculation of Carbon Emissions Embodied in Inter-Provincial Trade

**Figure 1:**
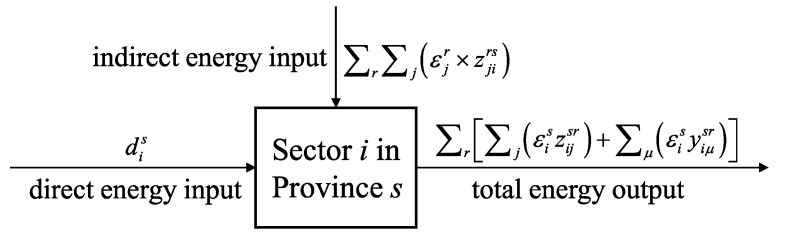
Schematic diagram of the embodied energy balance principle for sector *i* in province *s*.

We introduce emission intensity $\varepsilon_{i}^{s}$, which represents the total direct and indirect carbon emissions of a unit of goods or services produced by sector *i* in province *s*. According to the conservation law, the isolated system is always constant [37]. The embodied energy balance of sector *i* in province *s* is shown in Figure 1, which can be expressed as:

(12) $$d_{i}^{s}+\sum_{r}\sum_{j}\left(\varepsilon_{j}^{r}\times z_{ji}^{rs}\right)=\sum_{r}\left[\sum_{j}\left(\varepsilon_{i}^{s}z_{ij}^{sr}\right)+\sum_{\mu }\left(\varepsilon_{i}^{s}y_{i\mu }^{sr}\right)\right]$$

(13) $$d_{i}^{s}+\sum_{r}\sum_{j}\left(\varepsilon_{j}^{r}\times z_{ji}^{rs}\right)=\varepsilon_{i}^{s}x_{i}^{s}$$

Then the whole input–output table can be expressed in matrix form as:

(14) $$D+Z^{T}\times E=X\times E$$

According to the construction rules of the input–output table, it is known that $\left(X-Z^{T}\right)$ is reversible. By transforming Equation (14) we can obtain:

(15) $$E={\left(X-Z^{T}\right)}^{-1}\times D$$

Based on input–output relationships derived in Section 2.1, the embodied carbon emission transfers by sector within province *s* can be calculated as:

(16) $$F^{ss}=diag\left(E^{s}\right)\times {\left(I-A^{ss}\right)}^{-1}\times diag\left(Y^{ss}\right)$$

The calculation of embodied carbon emission transfers from province *s* to province *r* is:

(17) $$F^{sr}=diag\left(E^{s}\right)\times {\left(I-A^{ss}\right)}^{-1}\times diag\left(A^{sr}X^{r}+Y^{sr}\right)$$

### 2.3. Construction of the ECETN

Complex network theory and its metrics provide a new perspective for us, making it possible to analyze the characteristics of the network from a global viewpoint [35,38]. It transforms the inter-provincial embodied carbon emission transfer relationships into a network consisting of nodes and connected edges, where nodes denote provinces and edges are used to represent the embodied carbon emission transfer relationships between provinces. The inter-provincial embodied carbon emission transfer relationships can be represented by set $G\left(t\right)=\left\{V\left(t\right),E\left(t\right)\right\}$, where G(t) denotes the ECETN for year *t*. The set $V\left(t\right)=\left\{v_{1}\left(t\right),v_{2}\left(t\right),...,v_{m}\left(t\right)\right\}$ represents the provinces in ECETN in year *t*. The set $E\left(t\right)={\left\{e_{sr}\left(t\right)\right\}}_{m\times m}$ represents the embodied carbon emission transfers between provinces in year *t*. $e_{sr}\left(t\right)$ represents the embodied carbon emission transfers from province *s* to province *r* in year *t*. We set the total embodied carbon emission transfer from province *s* to province *r* in year *t* as $f^{sr}\left(t\right)$, which is calculated as:

(18) $$f^{sr}\left(t\right)=\sum_{i}\sum_{j}f_{ij}^{sr}\left(t\right)$$

where $f_{ij}^{sr}\left(t\right)$ is an element in matrix $F^{sr}\left(t\right)$. If $f^{sr}\left(t\right)>0$ then $e_{sr}\left(t\right)=1$, the weight of the edge is $w_{sr}\left(t\right)=f^{sr}\left(t\right)$; otherwise $e_{sr}\left(t\right)=0$, that is:

(19) $$e_{sr}\left(t\right)=\left\{\begin{array}{c} \begin{array}{c} 0,\mathrm{if}\;\mathrm{there}\;\mathrm{is}\;\mathrm{no}\;\mathrm{embodied}\;\mathrm{carbon}\;\mathrm{emission}\;\mathrm{transfer}\;\mathrm{from}\;\mathrm{province}\;s\;\mathrm{to}\;\mathrm{province}\;r. \end{array}\\ \begin{array}{c} 1,\mathrm{if}\;\mathrm{there}\;\mathrm{is}\;\mathrm{embodied}\;\mathrm{carbon}\;\mathrm{emission}\;\mathrm{transfer}\;\mathrm{from}\;\mathrm{province}\;s\;\mathrm{to}\;\mathrm{province}\;r \end{array}. \end{array}\right.$$

The above steps allow us to construct a directed weighted network for each studied year to describe its embodied carbon emission transfers in inter-provincial trade.

### 2.4. Indicators for Analyzing the ECETN

In this section, we analyze the ECETN using the complex network theory metrics, which are detailed below.

#### 2.4.1. Centrality Analysis

Centrality reflects the status and role of each node in the network [39]. In this paper, we use degree centrality, betweenness centrality, closeness centrality and eigenvector centrality to evaluate.

(1) Degree Centrality

In a network, the interconnectedness of the nodes gives them the ability to influence each other. Therefore, we use the degree of each province to show its importance. In the directed network, the degree of a node can be classified as degree-out and degree-in. The degree-out of the node *i* refers to the number of edges that point from the node *i* to other nodes, the degree-in of the node *i* refers to the number of edges that point to the node *i* from other nodes. They denote the number of embodied carbon outflow and inflow relationships in ECETN, respectively. The expressions are calculated as:

(20) $$k_{i}^{out}=\sum_{j}e_{ij}$$

(21) $$k_{i}^{in}=\sum_{j}e_{ji}$$

where $k_{i}^{out}$ and $k_{i}^{in}$ are the degree-out and degree-in of node *i* in ECETN, respectively.

In addition, we use the total weight of edges connected to the node to describe the strength. The higher the strength of the node, the stronger its influence. Similarly, in the directed network, the strength of a node can be divided into strength-out and strength-in. In ECETN, the strength-out reflects the total export of embodied carbon emissions of the node, and the strength-in reflects the total import of embodied carbon emissions of the node. The expressions are calculated as:

(22) $$s_{i}^{out}=\sum_{j}w_{ij}$$

(23) $$s_{i}^{in}=\sum_{j}w_{ji}$$

where $s_{i}^{out}$ and $s_{i}^{in}$ are the strength-out and strength-in of node *i* in ECETN, respectively.

(2) Betweenness Centrality

Betweenness centrality is defined by the number of shortest paths passing through the node, and it is used to measure the bridge properties and media capabilities of the node. In ECETN, provinces with high betweenness centrality are the key channels for controlling the embodied carbon emission transfers, they can absorb transfers from multiple provinces, and then transfer the embodied carbon emissions to other provinces. The normalized betweenness centrality of node *i* is calculated as:

(24) Bi=∑j≠l≠i(Ljli/Ljl)N−1N−2N−1N−222

where $L_{jl}$ is the number of all shortest paths from node *j* to node *l* that exist in the network, $L_{jl}\left(i\right)$ is the number of all shortest paths from node *j* to node *l* and passing through node *i*, and *N* is the number of nodes in the network.

(3) Closeness Centrality

The reciprocal of the sum of the shortest path lengths from the node to all other nodes is used to determine the closeness centrality. The greater the closeness centrality of a node, the closer it is to other nodes, and the lower the closeness centrality, the farther it is from other nodes. If the provinces with high closeness-out are affected somehow, the effect can quickly spread to other associated provinces through the shortest path. As a result, the effect can spread to most provinces or the entire network in the shortest possible time. For provinces with high closeness-in, if other provinces in the network are affected somehow, they can be affected in the shortest time. The closeness-out and closeness-in are calculated as:

(25) $$Cc_{i}^{out}={\left[\frac{1}{N-1}\sum_{j\neq i}d\left(i,j\right)\right]}^{-1}$$

(26) $$Cc_{i}^{in}={\left[\frac{1}{N-1}\sum_{j\neq i}d\left(j,i\right)\right]}^{-1}$$

where $d\left(i,j\right)$ is the shortest path length from node *i* to node *j*.

(4) Eigenvector Centrality

Eigenvector centrality considers that the importance of a node depends on both the number of its neighbors and the importance of its neighboring nodes. In ECETN, a node with huge embodied carbon emissions is definitely a critical node. However, if there is an embodied carbon emission transfer between a node and a critical node, that node can also be considered a relatively central node. Eigenvector centrality emphasizes the surroundings of the node, and nodes can increase their importance by connecting many other important nodes. The eigenvector centrality of node *i* is calculated as:

(27) $$Ec_{i}=\lambda^{-1}\sum_{j=1}^{N}e_{ij}\varepsilon_{j}$$

where λ and $\varepsilon_{j}$ are the maximum eigenvalue of the adjacency matrix and its eigenvector, respectively.

#### 2.4.2. Topology Analysis

(1) Network Density

The network density is defined as the ratio of the actual number of edges *M* to the maximum possible number of edges, which reflects the sparsity of the network. In the directed network its expression is defined as:

(28) $$\rho =\frac{M}{N\left(N-1\right)}$$

(2) Network Efficiency

The average shortest path length is usually used to measure the network efficiency. However, if there are multiple subgraphs in the network, the average shortest path between different subgraphs is infinite, which is not suitable for comparison. Therefore, network efficiency is achieved by calculating the reciprocal of distances between nodes, which is defined as:

(29) $$E=\frac{1}{N\left(N-1\right)}\sum_{i\neq j}\frac{1}{d_{ij}}$$

Network efficiency reflects the ease of embodied carbon emission transfer between the two provinces, that is, how many intermediate provinces embodied carbon emissions need to pass through before they are transferred to the target province. The higher the network efficiency, the faster the embodied carbon emissions are transferred to the whole network.

(3) Clustering coefficient

The clustering coefficient measures the probability that a node is connected to its neighbors, which evaluates the extent of nodes clustered together. If a node in the network has a high clustering coefficient, there is a close connection between it and its neighbors. Therefore, in ECETN, the clustering coefficient measures the relationship between the node and its trading nodes. In the directed weighted network, the clustering coefficient of a single node and the average clustering coefficient of the whole network are calculated as [40,41,42]:

(30) $$C_{i}=\frac{1}{s_{i}\left(k_{i}-1\right)}\sum_{j,h}\frac{w_{i,j}+w_{i,h}}{2}e_{i,j}e_{i,h}e_{j,h}$$

(31) $$C=\frac{1}{N}\sum_{i\in N}C_{i}$$

where $s_{i}$ is the strength of node *i*, and $k_{i}$ is the degree of node *i*. The average clustering coefficient of the network quantizes the connections between nodes in the network. If C=1, then all nodes are connected, while if *C* tends to 0, the network is more loosely connected.

(4) Assortativity

Assortativity considers the possibility of connecting nodes mainly from the perspective of network structure [43]. We use the assortativity coefficient (also known as Pearson Coefficient) to portray it, its expression is defined as:

(32) $$r=\frac{\sum_{i,j}\left(a_{ij}-\frac{k_{i}k_{j}}{2M}\right)k_{i}k_{j}}{\sum_{i,j}\left(k_{i}\delta_{ij}-\frac{k_{i}k_{j}}{2M}\right)k_{i}k_{j}},r\in \left[-1,1\right]$$

where $\delta_{ij}$ is the Kronecker delta. The essence of assortativity is the degree-degree correlation, which reflects the degree relationship between an interconnected pair of nodes. r>0 indicates that the entire network is assortative, and embodied carbon emissions tend to transfer to provinces with similar degrees. r<0 represents that the entire network exhibits disassortative, and embodied carbon emissions tend to transfer to provinces of different degrees. r=0 indicates that there is no obvious tendency for the connection between nodes, and the embodied carbon emission transfers exhibit stochasticity.

(5) Network Motif

The network may contain a variety of subgraphs, some of which occupy a significantly higher proportion than the proportion of these subgraphs in the corresponding random network, and these subgraphs are called motifs. Motif identification helps to identify typical local connectivity patterns in the network. To determine whether the subgraph *j* in the network is a motif, we can compare the occurrence number of the subgraph N(j) in the actual network with the average occurrence number $\langle N_{r}\left(j\right)\rangle$ in the corresponding random network, and we usually require $R\left(j\right)=\frac{N(j)}{\langle N_{r}\left(j\right)\rangle }>1.1$. The network motif allows to observe the local relationship patterns and inter-provincial interactions of embodied carbon emission transfers in ECETN.

#### 2.4.3. Clustering Analysis

We are more interested in whether the nodes in the network aggregate together in a certain way, in addition to knowing the fundamental statistics of the network. The block model is a method of partitioning nodes based on structural information. It is a method of studying the network location model and a descriptive algebraic analysis of social roles. The block model can partition a complex network into several sub-blocks to concisely describe the whole network. It can be used to profile the main connectivity modes and functional roles in the network. Block model analysis was first proposed by White in 1976 [44] and has since been further improved and generalized and gradually used to study some specific problems [41,45]. Based on Wasserman et al. (1994) [41] and Lv et al. (2019) [29], we can divide the network into four attribute categories: main inflow block, main outflow block, bidirectional spillover block, and agent block. The proportion of actual internal relationships is greater than the expected proportion of internal relationships in the main inflow block, receiving more contacts from other blocks. The number of external contacts is significantly higher than the number of internal contracts. The proportion of actual internal relationships in the main outflow block is smaller than expected. The external contacts received are significantly lower than the number of contacts sent. The bidirectional spillover block has a larger proportion of actual internal relationships, and it receives fewer contacts but sends more internal and external contacts. The agent block receives fewer contacts but sends more external and internal contacts. The proportion of actual internal relationships is smaller than the expected internal relationships. By explicitly examining how all blocks send and receive contacts with each other, we can perform a descriptive analysis of each block and ultimately achieve a study of the entire embodied carbon emission transfer system.

### 2.5. QAP Analysis

In ECETN, the structural role of provinces may have a certain impact on network performance. Therefore, we assume that degree-in, degree-out, strength-in, strength-out, closeness-in, closeness-out, betweenness centrality, eigenvector centrality, clustering coefficient, and spatial adjacency relationship of each province can affect the network. Based on the above analysis, we can build the model:

(33) $$G=f(K_{in},K_{out},S_{in},S_{out},Cc_{in},Cc_{out},B,Ec,C,SR)$$

All indicators in the model are in matrix form. *G* is the adjacency matrix of ECETN. $K_{in}$, $K_{out}$, $S_{in}$, $S_{out}$, $Cc_{in}$, $Cc_{out}$, *B*, Ec and *C* are the difference matrices constructed based on the degree-in, degree-out, strength-in, strength-out, closeness-in, closeness-out, betweenness centrality, eigenvector centrality, and clustering coefficient of all provinces, respectively. SR is the spatial adjacency relationship among provinces, and takes the value of 1 if two provinces are adjacent, and 0 if they are not.

In this study, there are certain relationships between matrices we choose for influence factor analysis, and the values of matrices are not independent of each other. Therefore, many traditional statistical analysis methods may lead to multicollinearity, making the calculated results have a large standard deviation and further affecting the results. In this paper, to better obtain credible results, we use QAP analysis to test the relationship between provincial roles and embodied carbon emission transfers and further analyze the impact of provincial roles on ECETN [46,47]. QAP analysis is a nonparametric test based on permutation, which tests the relationship between relational variables and does not focus on the overall distribution. Compared with the traditional parametric method, the QAP is more robust and does not require the assumption that the variables are independent of each other [48]. The QAP permutes the rows and columns of a given matrix simultaneously, which does not destroy the original data and ensures that the independent and dependent variable matrices are dependent on each other in rows and columns [49,50].

### 2.6. The Data

This study analyzes the spatial and temporal evolution of embodied carbon emissions from inter-provincial trade in 31 Chinese provinces (including provinces, municipalities, and autonomous regions, excluding Hong Kong, Macao, Taiwan, and the South China Sea islands). The inter-provincial trade input–output tables for 2012, 2015, and 2017 are taken from Carbon Emission Accounts & Datasets (CEADs) (https://www.ceads.net/data/input_output_tables, accessed on 14 May 2022), which is an essential database for researchers conducting input–output studies in China. It provides a complete set of input–output tables [51,52], including 42 sectors in 31 provinces. In particular, the input–output tables also provide the total carbon emissions of each sector [53,54,55,56], including coal, oil, natural gas, electricity, etc., which is crucial to our analysis of embodied carbon emissions.

## 3. Empirical Results

### 3.1. Evolution of Carbon Emissions Embodied in Inter-Provincial Trade in China

#### 3.1.1. Evolution of Embodied Carbon Emissions under the Provincial Perspective

**Figure 2:**
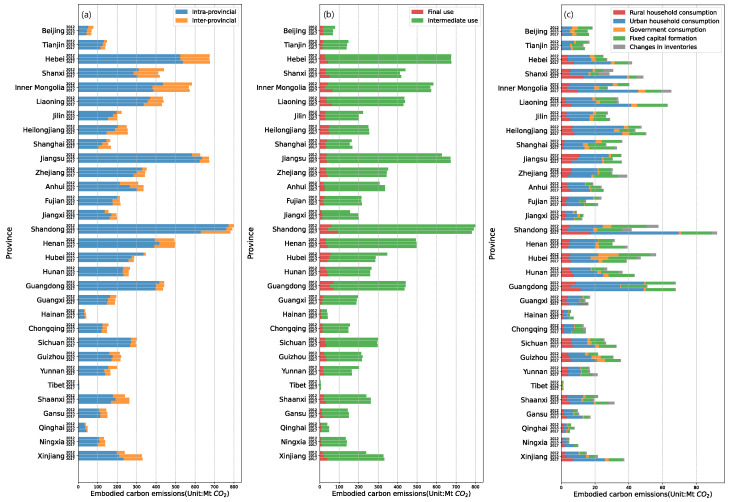
Embodied carbon emissions by province in China. (**a**) The scale of intra-provincial and inter-provincial embodied CO_2_ emissions in each province. (**b**) The scale of embodied CO_2_ emissions generated by intermediate use and final use in each province. (**c**) The scale of embodied CO_2_ emissions from different categories of final use in each province.

We calculate the embodied carbon emissions of 31 provinces using inter-provincial trade input–output tables of China for 2012, 2015, and 2017. The total scale of embodied carbon emissions in inter-provincial trade in China increased from 8889.36 Mt CO_2_ in 2012 to 8925.22 Mt CO_2_ in 2015 and to 8935.26 Mt CO_2_ in 2017. For each province, its embodied carbon emission scale in 2012, 2015, and 2017 is shown in Figure 2.

According to the scale of intra-provincial and inter-provincial embodied CO_2_ emissions of each province in Figure 2a, intra-provincial trade embodied CO_2_ emissions occupied the majority of total emissions, accounting for 84.34%, 82.69%, and 79.61% in 2012, 2015, and 2017, respectively, while inter-provincial trade embodied CO_2_ emissions accounted for a small share. In terms of embodied CO_2_ emissions, Hebei, Shanxi, Inner Mongolia, Liaoning, and Henan played an important role in inter-provincial embodied CO_2_ emissions. These provinces were all from the north and rich in coal, minerals, and other resources. Their energy needed not only to meet the development of their own province but also to export to other provinces, resulting in a significant inter-provincial transfer of embodied carbon emissions.

Based on the scale of embodied CO_2_ emissions generated by intermediate use and final use in each province in Figure 2b, we find that embodied CO_2_ emissions due to intermediate use accounted for the majority of total emissions, with the share of the total in 2012, 2015 and 2017 being 91.07%, 91.77%, and 88.80%, respectively. Shandong, Hebei, Jiangsu, Inner Mongolia, Shanxi, Liaoning, Henan, and Guangdong were the main direct carbon emission provinces, mostly located in northern China.

The scale of embodied CO_2_ emissions from different categories of final use in each province is shown in Figure 2c. In terms of household consumption, rural residential consumption accounted for 14.22%, 14.28%, and 15.84% in 2012, 2015, and 2017, respectively, and the corresponding urban residential consumption was 48.89%, 46.79%, and 48.63%, respectively. From 2012 to 2017, the Chinese urban population increased year by year, and on average, the urban and rural populations were similar in size, accounting for 55.67% and 44.33%, respectively. However, the embodied CO_2_ emissions of urban residential consumption were more than three times higher than those of rural areas. Therefore, in the process of urban and rural development, urban residential lifestyles need to be gradually improved and transformed into low-carbon consumption [4].

#### 3.1.2. Evolution of Embodied Carbon Emissions under the Sectoral Perspective

**Figure 3:**
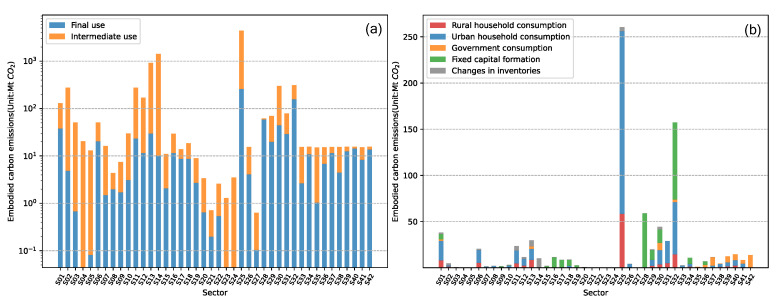
Embodied carbon emissions by sector in China. (**a**) The scale of embodied CO_2_ emissions from intermediate use and final use in each sector. (**b**) The scale of embodied CO_2_ emissions from different categories of final use in each sector.

The scale of embodied carbon emissions from intermediate use and final use in each sector is shown in Figure 3a. We find that the embodied carbon emissions from intermediate use in most sectors far exceed those from final use. The three sectors with the largest embodied carbon emissions are S13 (Manufacture of non-metallic mineral products), S14 (Smelting and processing of metals), and S25 (Production and distribution of electric power and heat power), accounting for 10.35%, 15.99%, and 49.76% of the total, respectively.

The scale of embodied CO_2_ emissions from different categories of final use in each sector is shown in Figure 3b. It can be seen that the embodied carbon emissions of S25 (Production and distribution of electric and heat power) and S32 (Information transfer, software, and information technology services) due to final use are high, accounting for 30.90% and 18.66% of the total, respectively.

### 3.2. Evolution of Structural Characteristics of ECETN

We construct ECETNs for 2012, 2015, and 2017, respectively. In this paper, we use inter-provincial trade input–output tables of China for our research. All provincial sectors consume energy to a greater or lesser extent and produce certain embodied carbon emissions so that the constructed ECETN is a fully connected network containing 31 nodes and 930 edges. Using complex network theory for network topology analysis in a fully connected network will reduce the validity of the results, making it difficult to reveal the structural characteristics of the network indeed.

**Figure 4:**
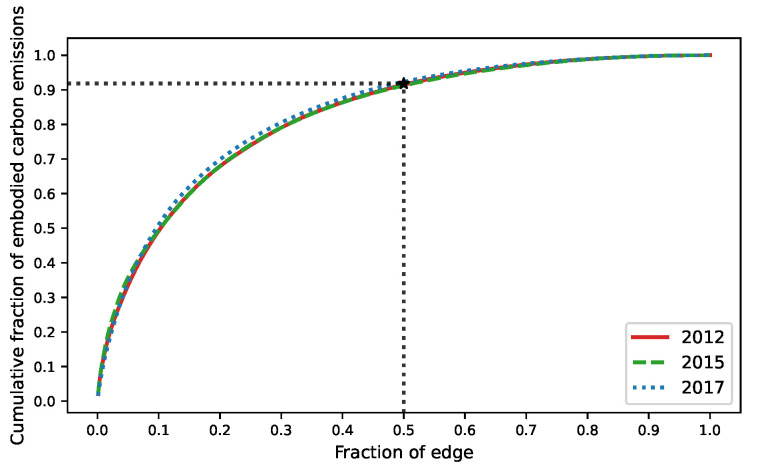
Cumulative fraction of edges by weight.

**Figure 5:**
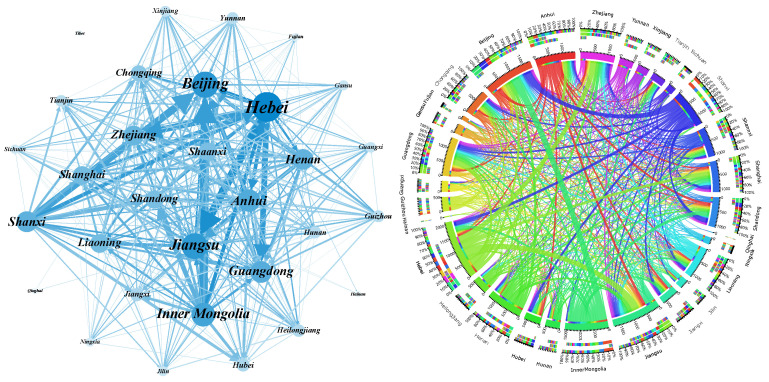
Inter-provincial embodied carbon emission transfers in 2012.

**Figure 6:**
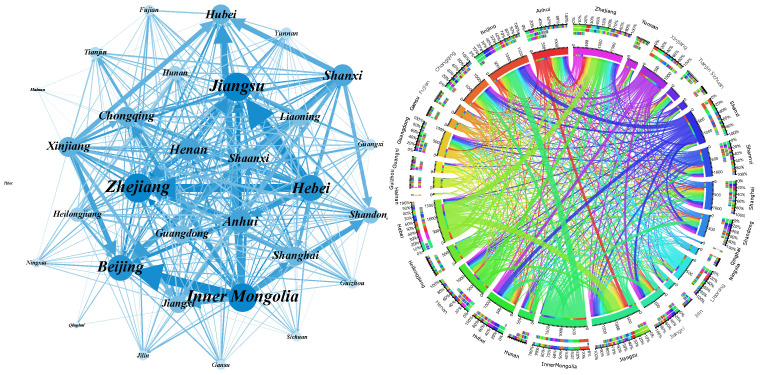
Inter-provincial embodied carbon emission transfers in 2015.

**Figure 7:**
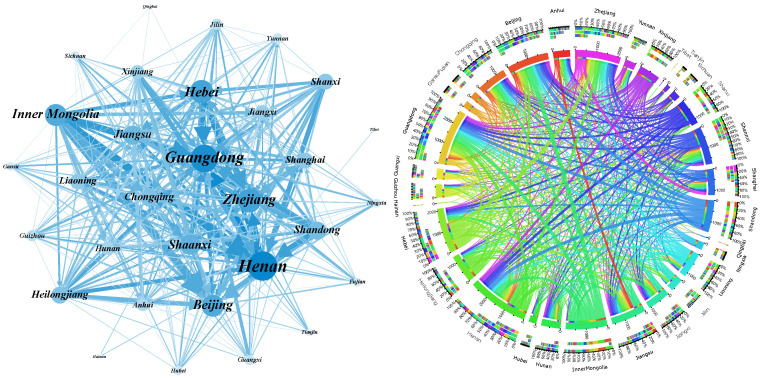
Inter-provincial embodied carbon emission transfers in 2017.

We rank all edges in the network according to the embodied carbon emission transfers from large to small and analyze the cumulative distribution of the weights. The results are shown in Figure 4. It is clear from the figure that 50% of the edges in the three original ECETNs in 2012, 2015, and 2017 carry more than 90% of the total weight of the whole network. To better highlight the important embodied carbon transfer relationships, we introduce a filtering procedure to keep the edges that occupy 90% of the weight of the original network and filter out the edges that occupy only 10% of the weight. We finally obtain the corresponding ECETNs for 2012, 2015, and 2017. To make the observation more intuitive, we visualize the network structure and its embodied carbon emission transfers, as shown in Figure 5, Figure 6 and Figure 7. In the figure, the left panel shows the network topology, and the right panel clearly shows the embodied carbon emission transfers and its scale between provinces. In the topology diagram, the size of the node indicates the total amount of embodied carbon transfers passing through the province, and the thickness of the edge indicates the amount of embodied carbon transfer between provinces. We find that the key provinces can be divided into two main categories: one is economically developed provinces, which are mainly importers of embodied carbon emissions, and the other is provinces rich in natural resources, which are mainly exporters of embodied carbon emissions. With the gradual development of transportation, geographical factors have less influence on trade, and the distribution of critical provinces becomes extensive.

We calculate the structural characteristic indicators of the ECETN corresponding to the three years using complex network theory, and the obtained results are shown in Table 2. As density decreases, the network becomes sparse. During the study period, the average shortest path decreases from 1.590 to 1.586 and then decreases to 1.560 in 2017, indicating that provinces are becoming more connected. It can also be observed that the number of edges and the average degree of the network gradually decrease while the average strength increases significantly, which indicates that the total amount of embodied carbon emission transfers is increasing. From the perspective of each province, the number of provinces with which there are significant embodied carbon emission transfer relationships gradually decreases, and embodied carbon emission transfers tend to concentrate in a few provinces. This performance can be confirmed in the trend of network efficiency. Nevertheless, these three networks still have characteristics of the high clustering coefficient and short average path length, which means they all have small-world characteristics, and the network performance is fragile and sensitive. Changes in embodied carbon emissions of key provinces will rapidly affect most provinces and thus lead to changes in the whole network. This also means that energy conservation and emission reduction policies in key provinces will significantly reduce the carbon emission level of the whole country. In addition, the assortativity of all three networks is less than 0, indicating that the embodied carbon emissions tend to transfer among provinces with different node degree values.

**Figure 8:**
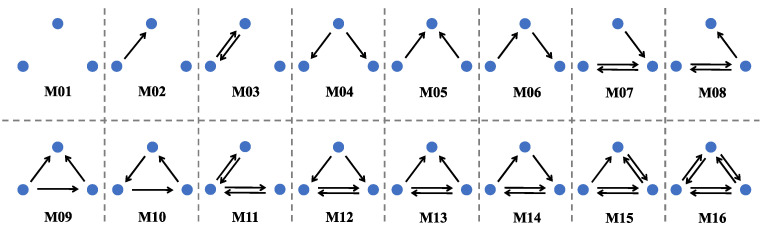
All possible interaction modes among the three nodes in the directed network.

**Figure 9:**
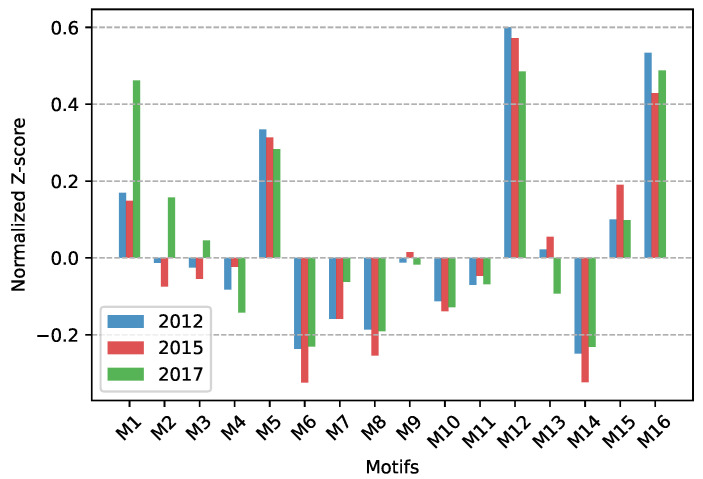
The TSP in ECETN.

The network motif can effectively reflect the local relationship pattern of the network [57]. We focus on the subgraph structure composed of 3 provincial nodes, and all possible interaction patterns among the 3 nodes in the directed network are shown in Figure 8. The statistical significance description for each network motif is usually achieved by a normalized *Z-score*, which is calculated as described in the study by Zeng et al. [35]. By calculating each subgraph, we finally get the Triad Significance Profile (TSP) in ECETN as shown in Figure 9, which reflects the importance of the 16 different triads in the network. It can be seen from the figure that the ECETNs in 2012 and 2015 show similar local relationship patterns, with higher significance levels for the motifs M5, M12, and M16. The importance of M1 significantly increases, and the importance of M5 and M16 decreases in the network in 2017.

### 3.3. Provincial Roles

In this subsection, we use different indicators to measure the importance of provinces from different perspectives. In the constructed ECETN for the study years, we calculate the centrality indicators for all provinces, and the calculation results are detailed in Table A1 in Appendix A.

#### 3.3.1. Provinces with Large-Scale Influence

Jiangsu, Zhejiang, Guangdong, and Beijing generally have a high degree-in in these three years, with an average of about 27, indicating that the embodied carbon emissions of about 27 of the 31 provinces studied are transferred to these provinces. These provinces with higher degree-in values relative to others can receive transfers from multiple provinces, resulting in large-scale influences on the entire network. If their consumer trade is controlled, the provinces that supply them will also be affected. We find that these provinces with larger degree-in values are generally provinces with a high level of domestic economic development, and most of them are in the south. These provinces require trade imports from most provinces due to their rapid development. It is worth noting that the degree-in values of Shanghai, Shandong, Hubei, and Tianjin show a significant downward trend in the three years, indicating that their import trade provinces are significantly reduced, showing a trend of concentration to a few provinces, while Henan, Hunan, and Yunnan show a significant increase in degree-in, indicating that they carry out trade imports from more provinces.

By observing the degree-out values of various provinces, we find that Hebei, Henan, and Inner Mongolia generally have higher degree-out in these years, with an average of about 24, indicating that the embodied carbon emissions of these provinces can be transferred to the other 24 provinces. These provinces conduct trade exports to multiple other provinces, thus affecting the entire network. If their trade exports are controlled, provinces importing from them will also be affected. It can also be seen that these provinces with high degree-out values are all in the north, are rich in resources, and export to most other provinces, resulting in a large amount of embodied carbon emissions transfers. In addition, we find that most provinces show a decreasing trend in degree-out values during these years.

Henan, Shaanxi, Hebei, Jiangsu, Zhejiang, and Guangdong have high degree values in ECETN, which shows that there are embodied carbon emission transfer relationships between these provinces and many other provinces, and there is a strong spatial correlation. Their trade and carbon emission policies can have a large scale of influence in the country, reflecting their relative importance. We distinguish degree-in, degree-out, and degree for analysis, making it possible to understand the degree of influence scale of each province with more precision and detail, which is a good reference for policymakers and planners.

#### 3.3.2. Provinces with Strong Influence

It can be seen from the table that Beijing, Zhejiang, Jiangsu, and Guangdong have higher strength-in values, which shows that other provinces transfer more embodied carbon emissions to them. It also reflects that these provinces have higher import trade demand from other provinces due to economic development. Beijing is the main port of China. Guangdong, Jiangsu, and Zhejiang are the three wealthiest provinces. Due to the scarcity of energy resources, they need to import large amounts of energy and energy-intensive products from other provinces. The strength-in of Tianjin, Shandong, and Hubei decrease significantly, indicating an increased level of self-sufficiency within their provinces and a decrease in import trade from other provinces. The strength-in of Henan, Hunan, Guangdong, Chongqing, Guizhou, and Shaanxi increases significantly, indicating that the higher development growth rate in these provinces requires higher import trade from other provinces.

The strength-out of Inner Mongolia, Hebei, and Shanxi far exceeds that of the other provinces, implying that they have the strongest influence on the other provinces in the network. In China, coal accounts for the largest share of total energy consumption, and the carbon emissions from mining and washing of coal are also significant. These three provinces are also rich in coal resources, have more fundamental industries, supply much energy to support the growth of other provinces, and export much energy, resulting in high embodied carbon emission transfers. Generally speaking, the strength-out of most provinces still shows a rising trend year by year, among which Heilongjiang, Jilin, Liaoning, Shandong, and Hubei are more prominent. It can be seen from this that the rapid development of China’s economy is still inseparable from the support of coal, minerals, and other resources.

In terms of the strength values of the provincial nodes, Hebei, Henan, Beijing, and Inner Mongolia far exceed the other provinces, while Zhejiang, Jiangsu, and Guangdong are slightly lower. Embodied carbon emissions transfer to and from these provinces is high, and they are the core provinces in the entire network. The embodied carbon emission transfer strength in Tibet, Qinghai, Hainan, and Ningxia is consistently low. On the one hand, they are located in remote areas with generally underdeveloped economies and low levels of industrial development. On the other hand, they are not responsible for major energy production, resulting in less embodied carbon emission transfers from inter-provincial trade products.

#### 3.3.3. Provinces with Strong Intermediary Ability

Henan, Hebei, Shaanxi, Jiangsu, and Zhejiang have high betweenness centrality. Nodes with high betweenness centrality generally maintain close contact with other provinces and firmly control the whole network. Therefore, these provinces should coordinate and communicate with each other to establish regional coordinated emission reduction strategies. Henan has a particular geographical location in China and also plays a key role as a bridge in the network, so special consideration should be given to Henan Province when formulating policies. Betweenness centrality rankings for most provinces do not change much over the years studied. Notably, the betweenness centrality indicators of Shandong, Anhui, and Inner Mongolia decrease significantly, and their intermediary role in the network gradually weakens. Shandong and Anhui have almost or even no intermediary role at all. In contrast, the betweenness centrality indicators of Xinjiang and Guangdong gradually increase and eventually play an essential intermediary role in ECETN of 2017. Hainan, Tibet, Gansu, Qinghai, and Ningxia have the lowest betweenness centrality. They are geographically remote, have weak economic foundations, and are technologically backward, so they are on the fringes of the network and are controlled by other provinces.

#### 3.3.4. Provinces with Strong Central Ability

Jiangsu, Zhejiang, Guangdong, and Beijing all have higher closeness-in values in the years studied, suggesting that the embodied carbon emission transfers from all provinces to them are more closely related and they are more susceptible to other provinces. It can be seen that the closeness-in of Shanghai, Anhui, Hubei, and Shandong gradually decreases, which means that the embodied carbon emission transfer relationships of other provinces to them gradually becomes looser. Henan, Hunan, Shaanxi, and Jiangxi gradually increase the value of closeness-in and become more and more closely linked to other provinces. Among the years studied, Henan and Hebei have high closeness-out values, and their own changes propagate rapidly throughout the network. Xinjiang, Shandong, and Shanghai show a remarkable increase in closeness-out and gradually become new centers in the network. At the same time, Anhui, on the contrary, exhibits a rapid decline in its closeness-out.

#### 3.3.5. Provinces with High Eigenvector Centrality

Henan, Shaanxi, and Hebei have high eigenvector centrality indicators, and they consistently rank in the top three over time through embodied carbon emission transfers with several core provinces. In the years under study, eigenvector centrality for Shanghai gradually increases, illustrating that its embodied carbon emission transfer contacts with core provinces are gradually increasing, with more frequent trade exchanges and a larger trade scale. On the contrary, the indicator of Anhui gradually decreases, indicating that the transfers between Anhui and the core provinces gradually decrease. Therefore, when formulating national emission reduction policies, it is necessary to consider the direct economic impact and the indirect economic impact.

### 3.4. Clustering and Spatial Spillover Structure Characteristics

We binarize the adjacency matrix of ECETN and use the Convergent Correlation (CONCOR) method to analyze the spatial clustering of the embodied carbon emission transfers. To ensure the best clustering effect, we set the maximum segmentation depth to 2 and the convergence criterion to 0.2, according to the practice of some scholars [29,50,58,59]. This can divide the studied provinces into four blocks, calculate the subgroup network density, and perform clustering analysis on the blocks according to their characteristics. It is worth noting that, through our observation, there is almost no embodied carbon emission transfer between Tibet and other provinces in the three networks studied. When blocking all 31 provinces, Tibet is always divided into a separate block, which seriously affects blocking effectiveness. Therefore, we classify Tibet as an isolated block according to its connections with other provinces and remove Tibet from the network when we block it.

The block division and spillover effect analysis in the corresponding ECETN for 2012, 2015, and 2017 are shown in Table 3. These provinces in Block I and Block II are generally more economically developed provinces in the eastern and central parts of China and are divided into two blocks due to their respective higher intra-block contacts. The rapid economic development of these provinces is accompanied by increased demand for trade with other provinces, especially the need to obtain energy resources, leading to a large-scale transfer of embodied carbon emissions to them. These provinces in Block III play the role of intermediary and bridge in ECETN. For example, Henan and Hebei mainly undertake the industrial transfer from Beijing, Tianjin, and the developed eastern provinces. There are many provinces in Block IV, and the north has rich resources such as coal, oil, and natural gas. The northwestern provinces cover a large area, are sparsely populated, and ecologically fragile, making environmental management difficult, but also rich in mineral resources. The southwestern provinces are also rich in mineral resources, especially non-ferrous metal resources, accounting for nearly half of China’s total reserves. Due to the geographical constraints, the economic conditions of these provinces are relatively backward, and the export of mineral resources leads to a significant transfer of embodied carbon emissions.

In ECETN for 2015, Most of the provinces in Block I are the fusion of the provinces in Block I and Block II in 2012. Due to the rapid construction and development of the transportation network, accessibility is significantly improved, resulting in an increase in the number of provinces within the main inflow block and stronger connections between provinces. Block II and Block III are closely related to other blocks, respectively, and they determine the direction of the embodied carbon emission transfers of the whole network. By comparison, we find that the number of provinces in the main outflow block in 2015 is significantly reduced compared to 2012, and the provinces in the northeast and north, which are mainly reduced, change from the main outflow block to the bidirectional spillover block. This shows that during the year, the export volume of these provinces rich in mineral resources decreased, and the trade volume of imports from other provinces increased.

In ECETN for 2017, The provinces included in Block I do not change much, and the economically developed provinces are always in the main inflow block. The provinces within the Block III are relatively scattered, and each plays its own bridge role in different regions of China. The number of contacts sent from the Block IV to other blocks is significantly high.

**Figure 10:**
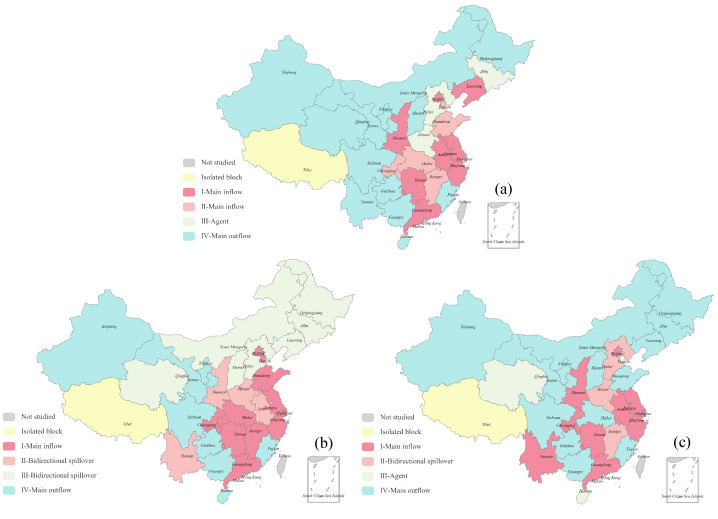
Block division of ECETN. (**a**) Composition of the blocks in China in 2012. (**b**) Composition of the blocks in China in 2015. (**c**) Composition of the blocks in China in 2017.

The block division in ECETN for the years studied is shown in Figure 10. As we can see from the figure, inter-provincial trade and its corresponding embodied carbon emission transfers are likely to appear in adjacent provinces due to geographical location, transportation costs, and trade demands, which can be referred to as geographical dependence [29]. To further analyze the spatial correlation of each divided block, the density matrix and the image matrix of block clustering in ECETN are calculated as shown in Table 4. The image matrix can visually reflect the spillover effect of embodied carbon emissions and clearly show the conduction mechanism between blocks. In general, the embodied carbon emission is transferred mainly to the economically developed eastern and central provinces in 2012, mainly to the eastern and southern provinces in 2015. The distribution of major transfer inflow provinces in 2017 is more dispersed but still concentrated in the eastern and southern regions. It can be seen that with the rapid economic development of the provinces, the demand for energy use increases, and the eastern and central provinces find it challenging to achieve self-sufficiency and need to rely on the energy-rich northern provinces for energy supply. At the same time, provinces in the Yangtze River Delta and Pearl River Delta regions have shifted their high-energy-consuming and high-polluting industries to their neighboring provinces with weaker economic development due to improved industrial structure and higher carbon emission standards. Therefore, the Chinese government and relevant departments should formulate corresponding policies and strengthen interregional cooperation and exchanges, which can effectively solve the problems of disharmony and disunity caused by objective factors such as geographical location.

### 3.5. Analysis of Influencing Factors of ECETN

We used 5000 random permutations to perform correlation analysis on the possible influences of ECETN, and the results of the analysis are shown in Table 5. Through correlation analysis, we find that strength-in difference, strength-out difference, betweenness centrality difference, and spatial adjacency always exhibit positive effects. The positive correlation coefficient of the spatial adjacency of provinces indicates that the embodied carbon emissions tend to be transferred between adjacent provinces. The differences in eigenvector centrality and clustering coefficient always show a significant negative effect, which reflects that the similarity of eigenvector centrality and clustering coefficient promotes the transfer of embodied carbon emissions between provinces.

We select the significant influencing factors in the correlation analysis as explanatory variables. We also use 5000 random permutations for the QAP regression analysis, and the regression results of ECETN with each explanatory variable are shown in Table 6. In general, the six explanatory variables of strength-in difference, strength-out difference, betweenness centrality difference, eigenvector centrality difference, clustering coefficient difference, and spatial adjacency relationship can always have a significant impact on ECETN. It is worth noting that the positive influence of the strength-in difference is the largest, the negative influence of the eigenvector centrality difference is the largest, and the positive influence of the spatial adjacency relationship gradually increases. This shows that the larger the difference in strength-in between provinces, the larger the embodied carbon emission transfers; the more similar the eigenvector centrality between provinces, the larger the scale of the embodied carbon emissions transfers. Geographically contiguous provinces are increasingly prone to transfer embodied carbon emissions, and embodied carbon emission transfers between non-contiguous provinces are decreasing. Our analysis is crucial in establishing a link between provincial roles and embodied carbon emissions transfers.

## 4. Conclusions and Policy Implications

Based on the empirical results, we can draw the following conclusions. First, The rapid economic development led to increased CO_2_ emissions from 2012 to 2017, and the total scale of embodied carbon emissions continued to rise. Second, The share of embodied CO_2_ emissions in inter-provincial trade increased from 13.66% in 2012 to 17.31% in 2015 and 20.39% in 2017. Third, the density of ECETN continued to decrease from 0.467 in 2012 to 0.445 in 2017, indicating that the network gradually became sparse. On the contrary, the scale of the embodied carbon emission transfers gradually increased, showing that from the perspective of each province, the transfer of embodied carbon emission tended to concentrate in a few provinces. The ECETN maintained a high clustering coefficient and a small average path length, implying that this network was sensitive and changes in carbon emission policies in some provinces could quickly affect the whole network. Fourth, provinces play different roles in the ECETN and exhibit different levels of importance. Fifth, the embodied carbon emissions show obvious regional disequilibrium characteristics. Sixth, by quantifying the contribution of provincial structural roles to the transfer of embodied carbon emissions, we find that strength-in difference, strength-out difference, betweenness centrality difference, and spatial adjacency have significant positive effects, and eigenvector centrality difference and clustering coefficient difference have significant negative effects.

Based on the findings of this paper, we can draw the following policy implications. First, we should focus more on reducing embodied CO_2_ emissions. The continuous expansion of the transfer of embodied CO_2_ emissions increases the differences in CO_2_ emissions between provinces. Resource-rich provinces can formulate strict emission policies to promote energy use efficiency and industrial structure upgrading to reduce carbon emissions. Second, provinces can develop different emission reduction plans according to their different roles in ECETN. Jiangsu, Zhejiang, Guangdong, Beijing, and other central provinces in the network should take the lead in reducing emissions and provide financial and technical support. Henan, Hebei, and Shaanxi as bridges can promote the diffusion of advanced technology among different subgroups in the network. Economically backward provinces such as Qinghai, Gansu, Ningxia, and Hainan need to increase network participation and accelerate the deployment of advanced CO_2_ emission control technologies. Third, considering the dependence of provinces on the transfer of embodied carbon emissions, China can implement a coordinated cross-provincial emission reduction strategy. The spatial aggregation effect of embodied carbon emission transfers is noticeable, and coordinated emission reduction policies can be implemented for provinces with the same spillover effect. Provinces that play the same role in the network can join forces to impose stricter regulations on carbon emissions at the source, promoting substantial reductions across the country. Fourth, China is now rich in renewable energy, with leading new energy technologies and industry scale at the forefront of the world. We need to increase the proportion of clean energy and renewable energy to achieve green production and consumption.

With rich research content and multiple perspectives, this study analyzes the spatial and temporal evolution characteristics of carbon emissions embodied in inter-provincial trade in China from the perspective of the system, region, and province, assisting policymakers in developing more effective and targeted national and provincial emission reduction policies. In contrast to the existing literature, this paper is the first to incorporate the topological characteristics of individuals into the analysis of the drivers. It is helpful to fully understand the effect of trade structure on embodied carbon emissions. This study provides a complete analytical framework for future embodied carbon emission analysis, which can also be used to study other embodied energy analyses of regional categories in different dimensions such as countries, provinces, cities, etc. The limitations of this paper are, first, our current work mainly focuses on static analysis; second, the uncertainty of the MRIO model itself leads to some errors in the analysis results [60]; and third, our analysis is based on the total trade scale of provinces, without further fine-grained studies on the impact of different trade types. Given these limitations, we have three main directions for future research: first, to realize the dynamic simulation of carbon emission reduction by adjusting the network structure of ECETN; second, to realize a more accurate calculation of the embodied carbon emissions based on the MRIO model combined with appropriate approaches; third, carbon emissions from the production and consumption sides and carbon emissions from different sectors can be further distinguished for more profound studies to determine a more comprehensive embodied carbon emission transfer relationship and further promote carbon emission reduction in China. 

## Figures and Tables

**Table 1:** Chinese MRIO table.

Input			Output															
			**Intermediate Use**							**Final Use**								**Total Output**
			**Province 1**			⋯	**Province** ***m***			**Province 1**			⋯	**Province** ***m***			**Exports**	
			**Sector 1**	⋯	**Sector** ***n***		**Sector 1**	⋯	**Sector** ***n***	**Category 1**	⋯	**Category** ***k***		**Category 1**	⋯	**Category** ***k***		
**Intermediate input**		Sector 1	$z_{11}^{11}$	⋯	$z_{1n}^{11}$		$z_{11}^{1m}$	⋯	$z_{1n}^{1m}$	$y_{11}^{11}$	⋯	$y_{1k}^{11}$		$y_{11}^{1m}$	⋯	$y_{1k}^{1m}$	$e_{1}^{1}$	$x_{1}^{1}$
	Province 1	⋮	⋮	⋱	⋮	⋯	⋮	⋱	⋮	⋮	⋱	⋮	⋯	⋮	⋱	⋮	⋮	⋮
		Sector *n*	$z_{n1}^{11}$	⋯	$z_{nn}^{11}$		$z_{n1}^{1m}$	⋯	$z_{nn}^{1m}$	$y_{n1}^{11}$	⋯	$y_{nk}^{11}$		$y_{n1}^{1m}$	⋯	$y_{nk}^{1m}$	$e_{n}^{1}$	$x_{n}^{1}$
	⋮		⋮							⋮							⋮	⋮
		Sector 1	$z_{11}^{m1}$	⋯	$z_{1n}^{m1}$		$z_{11}^{mm}$	⋯	$z_{1n}^{mm}$	$y_{11}^{m1}$	⋯	$y_{1k}^{m1}$		$y_{11}^{mm}$	⋯	$y_{1k}^{mm}$	$e_{1}^{m}$	$x_{1}^{m}$
	Province *m*	⋮	⋮	⋱	⋮	⋯	⋮	⋱	⋮	⋮	⋱	⋮	⋯	⋮	⋱	⋮	⋮	⋮
		Sector *n*	$z_{n1}^{m1}$	⋯	$z_{nn}^{m1}$		$z_{n1}^{mm}$	⋯	$z_{nn}^{mm}$	$y_{n1}^{m1}$	⋯	$y_{nk}^{m1}$		$y_{n1}^{mm}$	⋯	$y_{nk}^{mm}$	$e_{n}^{m}$	$x_{n}^{m}$
	Imports		$I_{1}^{1}$	⋯	$I_{n}^{1}$	⋯	$I_{1}^{m}$	⋯	$I_{n}^{m}$	$I_{1}^{f,1}$	⋯	$I_{k}^{f,1}$	⋯	$I_{1}^{f,m}$	⋯	$I_{k}^{f,m}$		
**Value-added**			$v_{1}^{1}$	⋯	$v_{n}^{1}$	⋯	$v_{1}^{m}$	⋯	$v_{n}^{m}$									
**Total input**			$x_{1}^{1}$	⋯	$x_{n}^{1}$	⋯	$x_{1}^{m}$	⋯	$x_{n}^{m}$									
**Direct carbon emissions**			$d_{1}^{1}$	⋯	$d_{n}^{1}$	⋯	$d_{1}^{m}$	⋯	$d_{n}^{m}$									

**Table 2:** Calculation results for structural characteristic indicators of ECETN.

Year	Number of Node	Number of Edge	Average Degree	Average Strength	Network Density	Network Efficiency	Clustering Coefficient	Average Path Length	Assortativity
2012	31	434	14.000	40.404	0.467	0.864	0.657	1.590	−0.272
2015	31	432	13.935	44.844	0.465	0.861	0.620	1.586	−0.254
2017	31	414	13.355	52.871	0.445	0.824	0.663	1.560	−0.319

**Table 3:** Block division and spillover effects of ECETN.

Year	Block	Provinces	Contacts Received		Contacts Sent		Expected Internal Relationship	Actual Internal Relationship	Characteristic
			Inside	Outside	Inside	Outside			
2012	I	Beijing, Anhui, Hunan, Guangdong, Zhejiang, Liaoning, Jiangsu, Shaanxi (8)	54	139	54	79	24.14%	40.60%	Main inflow
	II	Tianjin, Chongqing, Shandong, Shanghai, Jiangxi, Hubei (6)	10	127	10	43	17.24%	18.87%	Main inflow
	III	Henan, Jilin, Hebei (3)	4	45	4	57	6.90%	6.56%	Agent
	IV	Fujian, Inner Mongolia, Guangxi, Hainan, Heilongjiang, Sichuan, Guizhou, Yunnan, Shanxi, Gansu, Qinghai, Ningxia, Xinjiang (13)	19	36	19	168	41.38%	10.16%	Main outflow
2015	I	Beijing, Tianjin, Hunan, Guangdong, Shandong, Shanghai, Chongqing, Zhejiang, Jiangsu, Jiangxi, Hubei (11)	73	184	73	41	34.48%	64.04%	Main inflow
	II	Shaanxi, Yunnan, Henan, Anhui (4)	12	73	12	70	10.34%	14.63%	Bidirectional spillover
	III	Liaoning, Qinghai, Heilongjiang, Inner Mongolia, Shanxi, Jilin, Hebei (7)	26	27	26	99	20.69%	20.80%	Bidirectional spillover
	IV	Hainan, Guangxi, Guizhou, Sichuan, Gansu, Fujian, Ningxia, Xinjiang (8)	7	30	7	104	24.14%	6.31%	Main outflow
2017	I	Beijing, Shanghai, Hunan, Guangdong, Anhui, Shaanxi, Chongqing, Zhejiang, Yunnan, Jiangsu (10)	79	168	79	46	31.03%	63.20%	Main inflow
	II	Jiangxi, Henan, Hebei (3)	5	66	5	52	6.90%	8.77%	Bidirectional spillover
	III	Hainan, Qinghai, Tianjin (3)	0	5	0	9	6.90%	0.00%	Agent
	IV	Heilongjiang, Hubei, Shandong, Guangxi, Liaoning, Inner Mongolia, Sichuan, Guizhou, Jilin, Shanxi, Gansu, Fujian, Ningxia, Xinjiang (14)	49	41	49	173	44.83%	22.07%	Main outflow

**Table 4:** Density matrix and image matrix of blocks in ECETN.

Year	Block	Density Matrix				Image Matrix			
		I	II	III	IV	I	II	III	IV
2012	I	0.964	1.000	0.750	0.125	1	1	1	0
	II	0.750	0.333	0.278	0.026	1	0	0	0
	III	0.875	0.833	0.667	0.538	1	1	1	1
	IV	0.788	0.821	0.564	0.122	1	1	1	0
2015	I	0.709	0.682	0.104	0.034	1	1	0	0
	II	0.977	1.000	0.536	0.375	1	1	1	0
	III	0.844	0.679	0.619	0.268	1	1	1	0
	IV	0.864	0.750	0.071	0.125	1	1	0	0
2017	I	0.878	0.800	0.000	0.157	1	1	0	0
	II	1.000	0.833	0.333	0.452	1	1	0	0
	III	0.267	0.111	0.000	0.000	0	0	0	0
	IV	0.929	0.976	0.048	0.269	1	1	0	0

**Table 5:** QAP correlation analysis results of ECETN.

Variable	2012		2015		2017	
	ObsValue	Significance	ObsValue	Significance	ObsValue	Significance
$K_{in}$	−0.024	0.237	−0.071 **	0.024	0.052 *	0.066
$K_{out}$	−0.044	0.257	0.047	0.205	−0.119 **	0.043
$S_{in}$	0.211 ***	0.000	0.261 ***	0.000	0.303 ***	0.000
$S_{out}$	0.221 ***	0.001	0.214 ***	0.001	0.106 *	0.089
$Cc_{in}$	0.109 **	0.048	−0.009	0.462	−0.066	0.190
$Cc_{out}$	−0.088	0.143	−0.021	0.413	0.141 **	0.040
*B*	0.203 ***	0.005	0.098 **	0.023	0.269 ***	0.002
Ec	−0.224 ***	0.000	−0.206 ***	0.000	−0.192 ***	0.001
*C*	−0.218 ***	0.000	−0.173 ***	0.000	−0.138 **	0.034
SR	0.065 **	0.032	0.033**	0.038	0.115 ***	0.000

* is significant at the 10% level; ** is significant at the 5% level; *** is significant at the 1% level.

**Table 6:** QAP regression analysis results of ECETN.

Dependent Variable	Inter Provincial Embodied Carbon Emission Transfer Network	2012		2015		2017	
		Standardized Coefficient	Significance (*p*-Value)	Standardized Coefficient	Significance (*p*-Value)	Standardized Coefficient	Significance (*p*-Value)
Influencing factors (difference matrix)	$K_{in}$	—	—	−0.073	0.414	0.131 **	0.032
	$K_{out}$	—	—	—	—	−0.107	0.154
	$S_{in}$	0.360 ***	0.000	0.329 ***	0.000	0.345 ***	0.000
	$S_{out}$	0.232 ***	0.000	0.182 ***	0.000	0.157 ***	0.001
	$Cc_{in}$	0.223 ***	0.005	—	—	—	—
	$Cc_{out}$	—	—	—	—	0.129	0.111
	*B*	0.184 ***	0.000	0.184 ***	0.000	0.197 ***	0.000
	Ec	−0.409 ***	0.000	−0.381 ***	0.000	−0.378 ***	0.000
	*C*	−0.187 ***	0.005	−0.266 ***	0.000	−0.108 **	0.033
	SR	0.075 ***	0.002	0.082 ***	0.007	0.091 ***	0.001
Determination cofficient	$R^{2}$	0.372		0.352		0.378	
	Adjusted $R^{2}$	0.364		0.343		0.370	

* is significant at the 10% level; ** is significant at the 5% level; *** is significant at the 1% level.

## Data Availability

The MRIO tables in China used in this study can be obtained by contacting the corresponding author, or can be downloaded from CEADs at https://www.ceads.net/data/input_output_tables (accessed on 14 May 2022).

## Appendix A

**Table A1:** Calculation results of the centrality indicators for each province.

Index	Province	Degree-In			Degree-Out			Degree			Strength-In			Strength-Out			Strength			Betweenness			Closeness-In			Closeness-Out			Eigenvector		
		2012	2015	2017	2012	2015	2017	2012	2015	2017	2012	2015	2017	2012	2015	2017	2012	2015	2017	2012	2015	2017	2012	2015	2017	2012	2015	2017	2012	2015	2017
1	Beijing	26	27	27	15	10	10	41	37	37	154.80	150.18	176.97	24.02	18.36	18.90	178.82	168.54	195.87	0.01847	0.01738	0.00409	0.938	0.968	0.968	0.714	0.600	0.667	0.312	0.275	0.293
2	Tianjin	18	19	3	8	5	6	26	24	9	38.04	40.91	3.84	11.42	7.68	14.15	49.47	48.59	17.98	0.00013	0.00034	0.00000	0.732	0.769	0.526	0.545	0.526	0.588	0.203	0.190	0.079
3	Hebei	21	12	21	26	26	22	47	38	43	61.44	20.89	77.55	148.95	148.43	133.51	210.39	169.32	211.06	0.14629	0.04326	0.09371	0.811	0.638	0.811	1.000	0.938	0.938	0.363	0.315	0.348
4	Shanxi	3	7	3	22	20	19	25	27	22	3.50	10.91	10.31	127.80	123.41	110.27	131.30	134.32	120.58	0.00043	0.00352	0.00072	0.484	0.556	0.526	0.882	0.789	0.833	0.210	0.222	0.195
5	InnerMongolia	7	5	8	24	26	20	31	31	28	10.55	5.49	12.59	144.93	185.40	182.26	155.48	190.89	194.86	0.04252	0.05272	0.00405	0.556	0.508	0.577	0.938	0.938	0.857	0.262	0.243	0.235
6	Liaoning	24	12	17	20	20	18	44	32	35	51.20	18.58	31.13	60.16	76.39	87.13	111.36	94.97	118.26	0.04974	0.01698	0.01507	0.882	0.638	0.732	0.833	0.789	0.811	0.343	0.271	0.318
7	Jilin	10	10	8	10	10	17	20	20	25	15.38	18.61	22.28	17.93	16.32	41.13	33.31	34.92	63.41	0.00225	0.00110	0.00094	0.588	0.612	0.577	0.566	0.600	0.789	0.151	0.160	0.212
8	Heilongjiang	8	6	11	18	20	18	26	26	29	15.95	8.65	30.30	39.98	61.77	101.67	55.93	70.42	131.97	0.00400	0.00502	0.00933	0.545	0.545	0.625	0.789	0.789	0.811	0.205	0.213	0.223
9	Shanghai	27	25	21	9	13	18	36	38	39	97.08	86.59	64.55	13.57	23.63	57.62	110.65	110.22	122.17	0.02031	0.00885	0.02412	0.968	0.909	0.811	0.625	0.638	0.811	0.280	0.304	0.328
10	Jiangsu	28	27	28	16	16	13	44	43	41	136.73	161.66	121.44	38.15	30.50	40.61	174.88	192.17	162.06	0.05356	0.04119	0.02200	1.000	0.968	1.000	0.732	0.682	0.714	0.334	0.327	0.313
11	Zhejiang	27	27	28	13	17	14	40	44	42	104.50	157.09	161.12	18.30	39.83	52.98	122.81	196.92	214.10	0.02832	0.04980	0.03457	0.968	0.968	1.000	0.682	0.698	0.732	0.301	0.332	0.325
12	Anhui	24	25	20	17	20	7	41	45	27	54.48	75.51	56.45	89.43	66.56	23.13	143.91	142.07	79.58	0.01913	0.04022	0.00000	0.882	0.909	0.789	0.750	0.750	0.612	0.321	0.353	0.205
13	Fujian	6	7	0	8	14	12	14	21	12	6.30	8.13	0.00	7.72	32.38	32.49	14.02	40.51	32.49	0.00050	0.00010	0.00000	0.536	0.577	0.000	0.536	0.652	0.667	0.101	0.169	0.104
14	Jiangxi	21	20	23	7	9	13	28	29	36	49.15	77.05	75.89	12.95	20.73	31.94	62.10	97.77	107.83	0.00031	0.00037	0.00445	0.789	0.789	0.857	0.500	0.566	0.698	0.199	0.216	0.274
15	Shandong	26	18	1	12	10	21	38	28	22	84.84	67.40	2.00	21.56	21.43	146.99	106.40	88.83	148.99	0.03678	0.00175	0.00029	0.938	0.750	0.462	0.652	0.600	0.882	0.298	0.222	0.196
16	Henan	18	26	27	25	25	23	43	51	50	29.52	60.13	183.69	98.48	79.11	101.84	128.00	139.23	285.53	0.06296	0.14758	0.16813	0.750	0.938	0.968	0.968	0.882	0.968	0.345	0.395	0.384
17	Hubei	22	23	7	3	3	10	25	26	17	55.43	111.61	11.30	3.88	4.43	23.27	59.31	116.04	34.57	0.00014	0.00202	0.00000	0.811	0.857	0.556	0.455	0.448	0.667	0.179	0.194	0.129
18	Hunan	16	23	24	16	11	11	32	34	35	21.58	40.72	71.12	30.64	23.98	19.03	52.22	64.70	90.15	0.00372	0.00309	0.00385	0.714	0.857	0.882	0.732	0.612	0.667	0.257	0.264	0.275
19	Guangdong	27	27	28	14	15	14	41	42	42	117.47	66.60	231.22	20.82	35.76	32.00	138.28	102.37	263.21	0.02069	0.03754	0.04068	0.968	0.968	1.000	0.698	0.667	0.732	0.313	0.314	0.323
20	Guangxi	8	8	9	16	15	13	24	23	22	10.22	11.42	15.78	28.21	32.08	32.99	38.43	43.51	48.77	0.00122	0.00602	0.00045	0.588	0.600	0.600	0.732	0.667	0.698	0.190	0.162	0.180
21	Hainan	1	3	1	2	3	1	3	6	2	1.18	2.75	1.54	1.59	2.72	1.40	2.78	5.47	2.94	0.00000	0.00000	0.00000	0.469	0.526	0.517	0.441	0.429	0.455	0.024	0.039	0.022
22	Chongqing	23	26	27	14	10	10	37	36	37	64.05	100.79	119.26	25.30	15.95	19.10	89.35	116.74	138.36	0.01424	0.00778	0.01053	0.857	0.938	0.968	0.698	0.577	0.652	0.291	0.268	0.290
23	Sichuan	4	7	7	15	13	12	19	20	19	4.20	10.31	11.50	23.18	17.86	24.92	27.38	28.17	36.42	0.00000	0.00010	0.00060	0.492	0.556	0.566	0.714	0.638	0.698	0.154	0.161	0.168
24	Guizhou	1	3	15	18	16	16	19	19	31	0.80	3.09	21.22	50.16	37.59	40.78	50.96	40.68	62.00	0.00014	0.00031	0.01399	0.462	0.517	0.682	0.769	0.682	0.789	0.156	0.152	0.265
25	Yunnan	9	13	18	16	13	10	25	26	28	8.35	17.85	30.10	42.18	26.46	21.12	50.53	44.31	51.22	0.00053	0.00135	0.00126	0.556	0.667	0.750	0.732	0.638	0.667	0.193	0.215	0.241
26	Tibet	0	0	1	0	0	0	0	0	1	0.00	0.00	1.03	0.00	0.00	0.00	0.00	0.00	1.03	0.00000	0.00000	0.00000	0.000	0.000	0.492	0.000	0.000	0.000	0.000	0.000	0.014
27	Shaanxi	21	21	26	22	24	18	43	45	44	46.15	47.05	87.62	58.70	67.52	88.42	104.85	114.58	176.05	0.04319	0.08824	0.03748	0.811	0.811	0.938	0.882	0.882	0.811	0.332	0.353	0.355
28	Gansu	3	4	0	15	14	13	18	18	13	2.50	3.55	0.00	32.26	29.66	30.27	34.76	33.21	30.27	0.00000	0.00000	0.00000	0.484	0.526	0.000	0.714	0.652	0.652	0.146	0.130	0.100
29	Qinghai	0	1	1	2	3	2	2	4	3	0.00	0.87	1.53	1.62	4.55	2.11	1.62	5.42	3.64	0.00000	0.00000	0.00000	0.000	0.353	0.000	0.435	0.462	0.448	0.016	0.026	0.016
30	Ningxia	1	2	1	14	14	12	15	16	13	0.98	2.42	1.98	23.27	25.46	32.42	24.26	27.89	34.40	0.00000	0.00000	0.00000	0.370	0.375	0.357	0.714	0.667	0.714	0.113	0.110	0.106
31	Xinjiang	4	3	3	17	22	21	21	25	24	6.16	3.36	3.70	35.39	114.24	94.52	41.54	117.61	98.21	0.00053	0.01530	0.03381	0.508	0.517	0.526	0.750	0.811	0.909	0.177	0.202	0.214
